# Improved PCR based methods for detecting *C9orf72* hexanucleotide repeat expansions

**DOI:** 10.1016/j.mcp.2016.06.001

**Published:** 2016-08

**Authors:** Elaine M. Cleary, Suvankar Pal, Tara Azam, David J. Moore, Robert Swingler, George Gorrie, Laura Stephenson, Shuna Colville, Siddharthan Chandran, Mary Porteous, Jon P. Warner

**Affiliations:** a South East Scotland Genetics Service, Western General Hospital, Edinburgh, EH4 2XU, United Kingdom; b Euan Macdonald Centre for MND Research, 49 Little France Crescent, Edinburgh, EH16 4SB, United Kingdom; c Anne Rowling Regenerative Neurology Clinic, University of Edinburgh, 49 Little France Crescent, Edinburgh, EH16 4SB, United Kingdom

**Keywords:** *C9orf72*, Repeat-primed PCR, Amyotrophic lateral sclerosis, Genetic testing

## Abstract

Due to the GC-rich, repetitive nature of *C9orf72* hexanucleotide repeat expansions, PCR based detection methods are challenging. Several limitations of PCR have been reported and overcoming these could help to define the pathogenic range. There is also a need to develop improved repeat-primed PCR assays which allow detection even in the presence of genomic variation around the repeat region. We have optimised PCR conditions for the *C9orf72* hexanucleotide repeat expansion, using betaine as a co-solvent and specific cycling conditions, including slow ramping and a high denaturation temperature. We have developed a flanking assay, and repeat-primed PCR assays for both 3′ and 5′ ends of the repeat expansion, which when used together provide a robust strategy for detecting the presence or absence of expansions greater than ∼100 repeats, even in the presence of genomic variability at the 3′ end of the repeat. Using our assays, we have detected repeat expansions in 47/442 Scottish ALS patients. Furthermore, we recommend the combined use of these assays in a clinical diagnostic setting.

## Highlights

• Flanking PCR across *C9orf72* repeat expansions up to 900 repeats has been performed.
• RP-PCR assays described for both 3′ and 5′ ends of the *C9orf72* repeat expansion.
• Robust detection of expansions by RP-PCR for mosaic samples.
• Limitations of RP-PCR described.

## 1 Introduction

A hexanucleotide repeat expansion (HRE) of a noncoding GGGGCC repeat within the Chromosome 9 open reading frame 72 (*C9orf72*) gene has been identified as a major cause of amyotrophic lateral sclerosis (ALS, MIM: 612069) and frontotemporal lobar degeneration (FTLD, MIM: 600274) [1], [2]. In the UK population, 7.5% of patients with FTLD and 8.1% of patients with ALS have *C9orf72* expansions greater than 32 repeats [3].

The threshold size range of pathogenic alleles has not been well defined, and often relies on the technical cut-off of detection by PCR based assays (30–50 repeats) [2], [4]. There is one report of a stable 70 repeat allele in an unaffected individual expanding in his offspring, but further studies are required to determine whether anticipation is associated with this repeat expansion [5]. To ascertain the minimal pathogenic repeat size, it is necessary to detect and accurately measure repeat sizes in small expansion carriers.

Historically, Southern blotting has been regarded as the gold standard method for detecting and sizing large repeat expansions such as in Fragile X syndrome. However, improvements in PCR based methods, particularly repeat-primed (RP-) PCR [6], has meant that clinical diagnosis can now be made using PCR methods alone. RP-PCR uses a locus-specific flanking primer along with a paired repeat primer that amplifies from multiple sites within the repeat, generating a characteristic ladder of fragments after capillary electrophoresis. In *C9orf72*, somatic mosaicism for repeat length in blood samples has been reported, and this can make accurate interpretation of Southern blots challenging, as well as making it difficult to predict any genotype-phenotype correlations with varying repeat size [1], [3], [7], [8]. For this reason, developing reliable and robust RP-PCR methods is important, and others agree that Southern blot results should be interpreted in conjunction with RP-PCR [3].

Within both research and diagnostic settings, it is desirable to have high-throughput, rapid PCR based tests which are highly accurate and do not require large amounts of input DNA. The challenges of PCR amplification of the 100% GC rich *C9orf72* HRE have been highlighted by a blinded international study which showed a wide variability in results obtained by different research laboratories using PCR methods [9]. Furthermore, the presence of variable deletions and insertions at the 3′ end of the HRE [10], can adversely affect the reliability of PCR assays targeting this region [11].

There are various ways in which PCR can be enhanced such as the addition of co-solvents such as dimethyl sulfoxide and betaine, modified Taq polymerase and alteration of cycling conditions [12]. Heat-pulse extension (HPE) PCR has been reported to successfully allow amplification of repetitive GC-rich sequences similar to *C9orf72* HRE, and so in this study we used these cycling conditions as a starting point to then optimise for these amplicons [12].

The objectives of this study were to develop a conventional flanking PCR assay which could amplify repeat alleles beyond the 50–70 limit reported in the literature, and to optimise RP-PCR assays for both ends of the repeat to ascertain whether there were greater than 100 repeats present. We also wanted to overcome the issues of the Renton et al. assay where the expansion is not detected by RP-PCR in cases with genomic variability adjacent to the HRE [11]. These assays were then used to screen for *C9orf72* HRE in ALS patients from the Scottish population.

## 2 Materials and methods

### 2.1 Patients and DNA samples

442 consecutive DNA samples obtained from patients with ALS who donated blood for research to the Scottish Regenerative Neurology Tissue Bank, and were phenotyped as part of the Scottish Motor Neurone Disease (MND) Register (between 1989 and 2015) were analysed. The diagnostic criteria used by the Scottish MND Register were the Modified World Federation of Neurology (1989–1994) or ‘El Escorial’ (1995 onwards) [13], [14]. Clinical diagnostic samples received to the South East Scotland Genetics Service for *C9orf72* testing from 2013 to 2016 were also used for assay development. In addition, positive control DNA samples derived from lymphoblast cell lines were obtained from Coriell Cell Repositories. The Institute of Neurology (UCL, Queen Square, London) shared positive control DNA derived from blood, from two short expansion (60–120 repeats) carriers.

### 2.2 Ethics

Ethical approval for research analysis of the Scottish Regenerative Neurology Tissue Bank samples affiliated to the Scottish MND register was obtained from the East of Scotland Research Ethics Service. NHS clinical diagnostic samples were consented for assay development.

### 2.3 Molecular testing

DNA was extracted from whole blood samples by phenol-chloroform, manual salting out, the Nucleon BACC3 genomic DNA kit (Tepnel Life Sciences), or Chemagic DNA blood kit (Perkin Elmer).

PCR reactions, in a total volume of 20 μl consisted of 0.8× Optimized DyNAzyme™ EXT buffer, 0.16 mM dATP, 0.16 mM dTTP, 0.56 mM dCTP, 0.56 mM dGTP, 1.8 M Betaine and 0.12 U/μl DyNAzyme™ EXT DNA Polymerase (ThermoFisher Scientific). For flanking PCR, primers were at 1.25 μM and 20 ng DNA was added. For RP-PCR, primer concentrations were: FAM labelled flanking, 0.5 μM; repeat, 0.25 μM and Tail R; 0.75 μM, and 200 ng DNA was added. PCR primers are listed in Table 1. PCR amplification was carried out on a Veriti^®^ thermal cycler (Life Technologies). Cycling conditions are shown in Table 2.

PCR products were separated by capillary electrophoresis using an ABI 3130xL with a 50 cm array (Life Technologies) with either Genescan™ LIZ600 or LIZ1200 size standard (Life Technologies). Data was analysed using GeneMarker^®^ software v2.4.0 (Soft Genetics). Alternatively, PCR products were separated on 0.8% UltraPure agarose (ThermoFisher Scientific) gels in TBE buffer with 100 bp DNA ladder (Promega) and 1 kb DNA extension ladder (Invitrogen).

For Sanger sequencing, either flanking PCR or an alternative 3′ RP-PCR was used (Table 1). PCR products were purified using Agencourt Ampure XP (Beckman Coulter), as per the manufacturer’s instructions, using a Biomek^®^ NX robot (Beckman Coulter). Sequencing was then performed using R6 primer and BigDye^®^ Terminator v3.1 (Life Technologies). Agencourt CleanSeq (Beckman Coulter) was used, according to the manufacturer’s instructions, to clean-up sequencing products prior to capillary electrophoresis on an ABI 3130xL (Life Technologies). Data was analysed using Mutation Surveyor^®^ software v4.0.8 (Soft Genetics).

## 3 Results

### 3.1 *C9orf72* HRE frequency in the Scottish ALS population

**Fig. 1:**
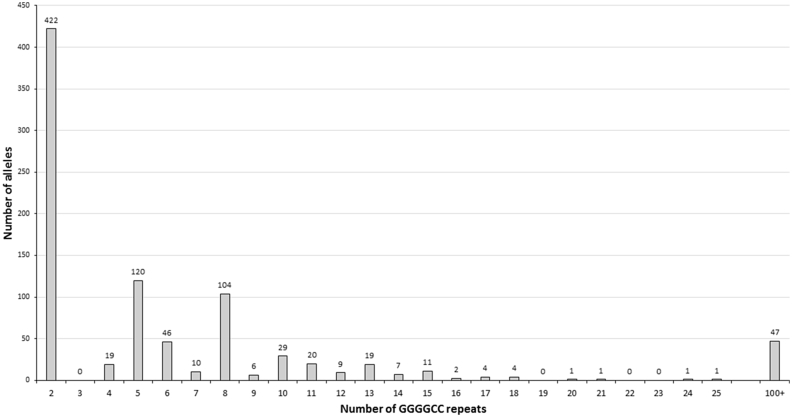
Size distribution of the (GGGGCC)n repeat in Scottish ALS patients. Histogram showing frequency of (GGGGCC)n repeat sizes in Scottish ALS patients, with expansions represented as 100 + repeats. Homozygous normal flanking results, where there is no expansion detected by RP-PCR have been counted twice to give allele frequency. No expansions between 26 and 100 repeats were detected.

We tested 442 archival DNA samples from the Scottish Regenerative Neurology Tissue Bank, linked to the Scottish MND Register, collected from 1989 to 2015, using flanking PCR to assess the sizes of normal alleles. 157 cases which gave a homozygous result on this assay were then tested using both 3′RP-PCR and 5′RP-PCR, which led to detection of *C9orf72* expansions in 47 patients (10.6%), and gave one equivocal result which could not be resolved due to insufficient DNA. The repeat sizes that were obtained are shown in Fig. 1, which shows a similar distribution to the UK population [3].

### 3.2 Optimal conditions for flanking PCR

**Fig. 2:**
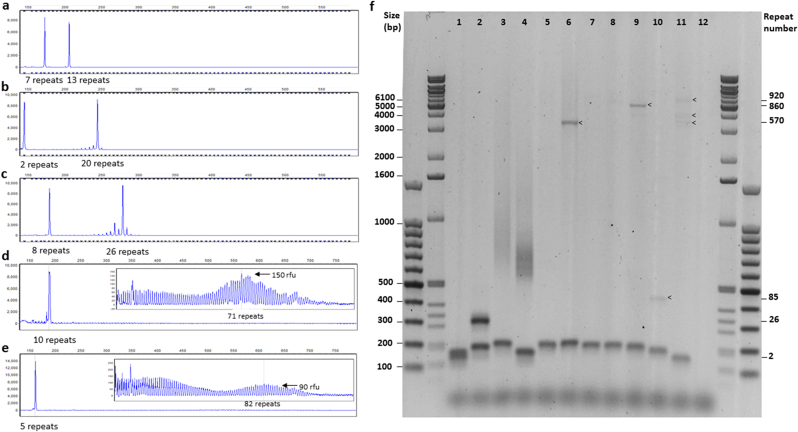
Flanking PCR results. Capillary electrophoresis traces following flanking PCR, showing relatively balanced amplification of (a) 7 and 13 repeat alleles, (b) 2 and 20 repeat alleles and (c) 8 and 26 repeat alleles, with some stuttering for the latter. (d) Capillary electrophoresis traces for a sample estimated to have 5; 60 repeats, with somatic mosaicism for a large expansion by Southern blot and (e) a sample estimated to have 5; 70–120 repeats by Southern blot [15]. All traces are from GeneMarker (Soft Genetics) and x axis corresponds to size in base pairs (bp) and y axis to relative fluorescent units (boxes in (d,e) show zoomed in trace of affected region (>30 repeats). (f) Agarose gel electrophoresis of flanking PCR products with 100 bp and 1 kb extension DNA ladders. Lanes 1–4 Blood derived DNA: 1. Normal control (2; 5 repeats). 2. Normal control (8; 26 repeats). 3. Expansion carrier (10; 60 mosaic repeats). 4. Expansion carrier; (5; 70–120 repeats). Lanes 5–11 Coriell Cell Repositories *C9orf72* HRE positive LCL DNA: 5. ND09373 (10; Expansion undetected). 6. ND09438 (11; 570 repeats). 7. ND10966 (9; Expansion undetected). 8. ND10973 (9; Expansion undetected). 9. ND11836 (8; 860 repeats). 10. ND12199 (6; 85 repeats). 11. ND14442 (2; 600,700,920 repeats). 12. No DNA control. Southern blot results [16] are consistent with a lack of amplification of the largest expanded material in cell line DNA.

We developed a PCR assay using primers flanking the *C9orf72* HRE and applied the HPE PCR conditions developed for Fragile X syndrome [12]. HPE PCR involves multiple heat pulses during the extension phase of the cycling protocol to temporarily destabilize GC rich structures which may otherwise lead to replication stalling [12]. These conditions permitted superior amplification to that achieved with Qiagen Multiplex PCR kit or Roche Fast Start High Fidelity PCR system with standard cycling conditions (data not shown). We then varied cycling conditions to determine the annealing temperature, and whether high denaturation, slow ramping or heat-pulse extension were required, and also the optimal extension time. We found that the slow ramp from annealing to extension phase and high denaturation temperature were the most important features, and in this case the heat pulses during extension were of no benefit (data not shown). The optimised conditions gave relatively balanced amplification of normal alleles, as highlighted in the series of samples with alleles ranging between 2 and 26 repeats (Fig. 2a–c). The Institute of Neurology, UCL, Queen Square, London sent us two samples with ‘short’ expansions. The first was estimated as having 60 repeats, with mosaicism for a large expansion (James Polke, personal communication), and another with 90 repeats in blood estimated by Southern blotting [15]. These alleles had not been amplified using existing PCR methods by ourselves or the Institute of Neurology (data not shown). Using our method, we could detect alleles of approximately 70 and 80 repeats, and revealed a high level of mosaicism in both cases (Fig. 2d–f). The largest repeat size we detected in blood was ∼120 repeats, although we did note that a large smear was present in a number of samples with expansions present (data not shown). To determine the upper size range of detection, we tested lymphoblast cell line DNA from the Coriell Cell Repository which was positive for *C9orf72* HRE by RP-PCR. This revealed material up to 5.7 kb, corresponding with approximately 900 repeats to have been amplified (Fig. 2f). There was amplification of expanded material in 4 out of 7 lines tested, and we presume that the other lines contained expansions which were beyond the size limit of detection by this method. This is supported by previously published Southern blotting results for ND10966, ND11836 and ND14442 [16].

To calibrate our sizing assay, we sequenced 14 patient samples with normal sized alleles to correlate the fragment size to repeat length. However, we cannot exclude variation in flanking sequences affecting the reported allele size, as has been reported by others [9].

### 3.3 Optimal conditions for RP-PCR

We designed primers for RP-PCR assays from both 3′ and 5′ ends of the HRE. We compared different PCR cycling conditions and found that the optimal conditions were the same as for flanking PCR, but with annealing at 62 °C.

**Fig. 3:**
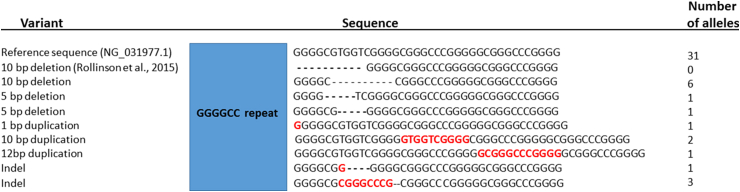
Sequence variants detected. An illustration of sequence variants detected at the 3′ end of the hexanucleotide repeat expansion. Deleted nucleotides are indicated as a dash (–) and insertions/duplications in red.

For the 5′ RP-PCR and 3′ RP-PCR assays, the maximum length of the amplicons which were obtained correlated with 100 and 160 repeats respectively. When including heat pulses [12] in the extension phase of the 3′ RP-PCR assay, we incidentally observed that there was a lack of amplification of normal alleles in the presence of an expansion, and used this assay to specifically sequence the expanded allele. This allowed us to investigate whether the optimised RP-PCR conditions permitted amplification even in cases which had variability in the 3′ end of the repeat, which has previously been reported to hamper PCR [11]. Out of 47 patients who tested positive for the expansion, we found that 31 of them had sequence which matched the reference sequence. This left a further 16 (34%) that had some form of insertion or deletion present at the 3′ end of the repeat, as shown in Fig. 3.

**Fig. 4:**
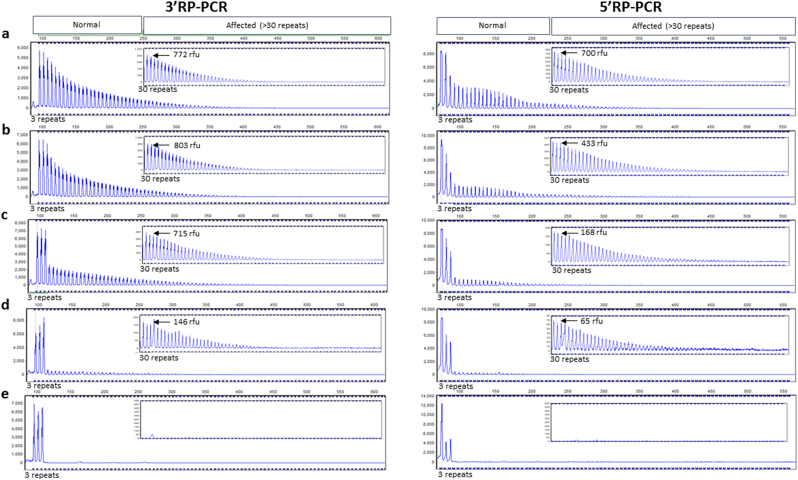
Sensitivity of RP-PCR for mosaicism. All capillary electrophoresis traces are of 3′RP-PCR (left) and 5′RP-PCR (right). The traces show amplification of heterozygous expanded DNA (2; Expansion) at varying degrees of dilution (a) 100% (b) 50% (c) 10% (d) 1% and (e) 0% in heterozygous normal control DNA (2; 5 repeats). Traces are from GeneMarker (Soft Genetics); the x axis corresponds to size in base pairs (bp) and y axis to relative fluorescent units (box shows zoomed in trace of affected region (>30 repeats).

A potential limitation of RP-PCR is preferential amplification of normal sized products preventing amplification of large expansions, and to investigate this issue we performed admixture experiments for both 3′RP-PCR and 5′RP-PCR assays. Dilution of a heterozygous expanded carrier, in a heterozygous normal control with 2 and 5 repeats, showed that both assays could still detect an expansion even when only present at 1%, as shown in Fig. 4.

**Fig. 5:**
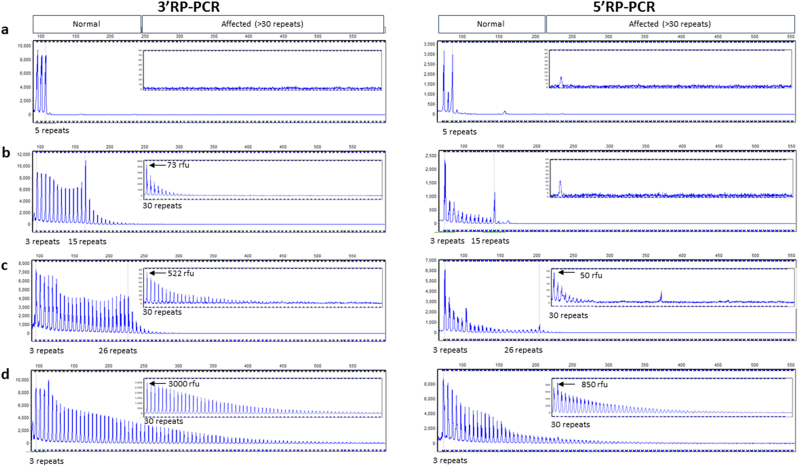
3′RP-PCR artefact present in samples with normal alleles greater than 15 repeats, which is not observed for 5′RP-PCR. All capillary electrophoresis traces are of 3′RP-PCR (left) and 5′RP-PCR (right). (a) Heterozygous normal sample (2; 5 repeats) (b) Heterozygous normal sample (2; 15 repeats) amplified with 3′RP-PCR showing weak stuttering into the affected range (522 rfu at 30 repeats), with 5′RP-PCR showing limited presence of product >30 repeats (50 rfu at 30 repeats). (c) Heterozygous normal sample (8; 26 repeats) amplified with 3′RP-PCR showing stuttering into the affected range, with 5′RP-PCR showing 10-fold weaker stuttering into the expanded range. (d) Heterozygous expansion carrier showing stronger signal in the affected (>30 repeat) size range in both assays compared to the samples with large normal alleles. Traces are from GeneMarker (Soft Genetics); the x axis corresponds to size in base pairs (bp) and y axis to relative fluorescent units (box shows zoomed in trace of affected region (>30 repeats).

For 156/157 homozygous normal patients tested, results for the 3′ and 5′ RP-PCR assays were concordant. The one discordant result was apparently homozygous for 15 repeats on flanking PCR, and only showed an expansion using the 3′ RP-PCR assay. Further analysis of heterozygous samples where one of the normal alleles was 15 repeats or longer revealed that the 3′ RP-PCR assay does not drop to the baseline after the larger normal allele peak, unlike the 5′ RP-PCR assay, as shown in Fig. 5. This effect is more pronounced the larger the normal allele is, as the stuttering is more likely to go into the affected range. Attempts to reduce this effect, by altering annealing temperature, reducing polymerase concentration and reducing cycle number failed to completely eliminate this PCR artefact, as they also resulted in an undesirable weaker trace for positive samples (data not shown).

## 4 Discussion

We have developed robust PCR based methods for detecting the HRE in *C9orf72*. The inclusion of betaine, along with *Taq* polymerase which is optimised for long, GC-rich regions and slow-ramping PCR cycling, all contribute to efficient PCR of this challenging genomic region. We have used our PCR methods to screen a cohort of 442 Scottish ALS patients for the HRE, as well in a clinical diagnostic setting for patients with ALS and FTLD.

The flanking PCR allows detection of alleles which are larger than have previously been reported using similar methods. Although the largest alleles were detected in cell line DNA which is not a source routinely used in a diagnostic setting, this gives an indication that the PCR is efficient and will be informative for blood samples with stable expansions of similar size. As much *C9orf72* research is performed on cell lines, this technique could be used to monitor repeat stability in culture. The detection of repeats in the 70–120 repeat range by PCR and capillary electrophoresis allows a more accurate size to be assigned as compared to agarose gel electrophoresis and Southern blotting, which has a lower resolution and is also non-denaturing so more affected by secondary structure formation [17].

For clinical diagnostic testing, it is important to be aware of the common repeat sizes within the population as this can guide testing. Suspicion arises when a patient is apparently homozygous for a rare repeat size, particularly if these are in the 15–30 range which could hamper PCR amplification [11]. In our experience, sequencing of the flanking PCR products in apparently homozygous cases can also reveal normal alleles with genomic variability, which has also been reported by others [9].

The RP-PCR assays that we have developed appear to be higher yielding and produce a ladder of fragments corresponding with over 100 repeats, which is longer than has previously been published [1], [2]. Importantly, our 3′ RP-PCR assay has been shown to be robust even in the presence of a number of genomic variations next to the HRE. We detected a relatively higher degree of variation than has been reported in studies based on Southern UK populations, with a 10 bp deletion being reported commonly in Northern England [11]. The prevalence of *C9orf72* expansions in the Scottish ALS population is similar to that reported in other population based series of ALS internationally [3], [18].

The admixture experiments which were carried out, where expansions can be detected even when diluted to 1% in a normal background, suggest that these assays are not severely affected by preferential amplification, and the conditions seem to be optimal for PCR of longer fragments. We have observed that in the 3′RP-PCR, normal alleles of greater than 15 repeats can lead to a PCR artefact with low level expanded material being observed. This presumed primer-product or product-product interaction, leading to replication slippage can only be reduced by measures which also reduce the production of expanded material in HRE positive cases. Thus, this may be a limitation for product length for *C9orf72* RP-PCR, as the 5′RP does not generate as large products and does not suffer from this artefact. The prevalence of false positive results in the study by Akimoto et al. [9] suggests that laboratories should be aware of such test limitations.

## 5 Conclusion

We would recommend testing using all three PCR assays in a clinical diagnostic setting, and ensuring there are concordant results prior to reporting. There may be rare cases (1/450 in this study) which are homozygous normal on flanking PCR and an expansion is only apparent in one RP-PCR assay, where reflex testing with Southern blotting may be necessary to obtain a result. Both RP-PCR assays should be used together to minimise the risk of any rare genomic variability, including single nucleotide polymorphisms under primer binding sites, from affecting the test accuracy. This is in line with recommendations for other repeat expansion disorders, such as Myotonic Dystrophy type 1 [19].

## Author contributions

RS, SP, MP, GG and SCh were involved in conception and running of the Scottish MND Register and provided clinical input. SCo and LS consented patients, collected and managed the samples. EMC and JPW designed the experiments, EMC performed the experiments, EMC, JPW, TA and DJM analysed the data and EMC wrote the manuscript. SP and JPW edited the manuscript.

## Figures and Tables

**Table 1:** Primer sequences.

Primer	Sequence (5′-3′)	Annealing temperature
Flanking PCR:		58 °C
F3	FAM-AGCAAG CTCTGG AACTCA GGAGTC G
R6	CCTCAC TCACCC ACTCGC CAC
3′ RP PCR:		62 °C
R8	FAM-CGGGCG CAGGCA CCGCAA CC
Repeat F3	TACGCA TCCCAG TTTGAG ACG**GGC CGGGGC CGGGGC CGG**
Tail R	TACGCA TCCCAG TTTGAG ACG
5′ RP PCR:		62 °C
F2	FAM-CTGTAG CAAGCT CTGGAA CTCAGG AGTCG
Repeat R	TACGCA TCCCAG TTTGAG ACG**CCC CGGCCC CGGCCC CGGCCC C**
Tail R	TACGCA TCCCAG TTTGAG ACG
3′RP PCR (sequencing):		61 °C
R6	CCTCAC TCACCC ACTCGC CAC
Repeat F	TACGCA TCCCAG TTTGAG ACG**GGG GCCGGG GCCGGG GCCGGG G**
Tail R	TACGCA TCCCAG TTTGAG ACG

**Table 2:** PCR cycling conditions.

94 °C	7 min	
95 °C	45 s	35 cycles
98 °C	10 s
58 °C (flanking) or 62 °C (RP-PCR)	30 s
78 °C (slow ramp 0.6 °C/s)	6 min
78 °C	10 min	
